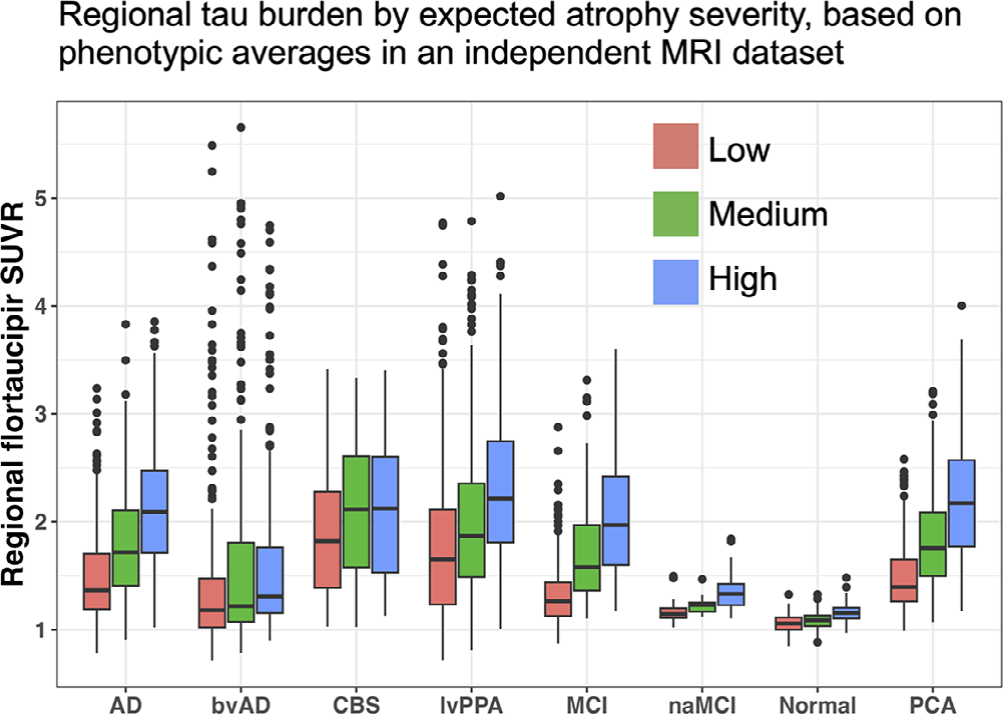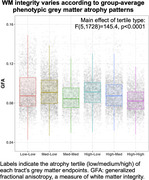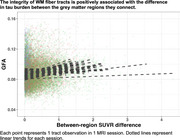# Non‐amnestic AD syndrome would exhibit WM differences from amnestic and other non‐amnestic syndromes

**DOI:** 10.1002/alz70856_097701

**Published:** 2025-12-24

**Authors:** Briar Nowling, Hamsanandini Radhakrishnan, Christopher A Olm, Philip A. Cook, Katheryn A. Q. Cousins, Leslie M. Shaw, Allison Snyder, David J. Irwin, Corey T. McMillan, Lauren Massimo, Ilya M. Nasrallah, Jeffrey S Phillips

**Affiliations:** ^1^ Frontotemporal Degeneration Center, University of Pennsylvania, Philadelphia, PA, USA; ^2^ Penn FTD Center, University of Pennsylvania, Philadelphia, PA, USA; ^3^ Penn Image Computing & Science Laboratory, Department of Radiology, University of Pennsylvania, Philadelphia, PA, USA; ^4^ Clinical Toxicology Laboratory, Perelman School of Medicine, University of Pennsylvania, Philadelphia, PA, USA; ^5^ Penn Frontotemporal Degeneration Center, Department of Neurology, Perelman School of Medicine, University of Pennsylvania, Philadelphia, PA, USA; ^6^ University of Pennsylvania, Philadelphia, PA, USA; ^7^ Centre for Biomedical Image Computing and Analytics, University of Pennsylvania, Philadelphia, PA, USA; ^8^ Penn Frontotemporal Degeneration Center, Department of Neurology, Perelman School of Medicine, University of Pennsylvania, Philadelphia, PA, USA

## Abstract

**Background:**

We have shown non‐amnestic Alzheimer's disease (AD) has greater white matter (WM) degeneration than amnestic AD. Understanding whether WM changes in AD are related to disease pathophysiology may be key to explaining the earlier onset and atypical tau distribution of non‐amnestic AD. We hypothesized WM degeneration would correlate with tau burden and atrophy in the grey matter endpoints of fiber tracts, linking WM change to tau progression.

**Method:**

We included 46 participants with normal cognition (*n* = 4) or autopsy, amyloid positron emission tomography (PET), or cerebrospinal fluid evidence of AD (*n* = 42). Clinical diagnoses included amnestic mild cognitive impairment (MCI) or AD (*n* = 15); or non‐amnestic syndromes including non‐amnestic MCI (*n* = 1); corticobasal syndrome (*n* = 3); posterior cortical atrophy (*n* = 6); logopenic‐variant primary progressive aphasia (*n* = 10); and behavioral/dysexecutive AD (*n* = 7). For each syndrome, we grouped brain regions into low, intermediate, and high atrophy based on syndrome‐specific atrophy patterns in *n* = 904 T1 MRI scans from separate individuals. Tau burden was assessed in 56 flortaucipir PET scans. We performed deterministic tractography on multishell, 96‐direction diffusion MRI acquired within 2 years of PET. WM integrity was measured using mean generalized fractional anisotropy (GFA) over each tract. Linear mixed effects models were used to contrast GFA and nodal degree between amnestic and non‐amnestic AD; and contrast GFA for tracts connecting high‐ vs. low‐atrophy regions. Finally, we tested associations between GFA and PET standardized uptake value ratio (SUVR) in tract endpoints.

**Result:**

GFA did not differ between groups, but nodal degree was lower in non‐amnestic AD (*p* = 0.024), indicating sparser connectivity. Across AD variants, tau SUVR increased (Figure 1) from lowest to highest atrophy tertile (*p* <0.0001). GFA was lower in tracts projecting from areas of higher atrophy than from less‐atrophied regions (Figure 2). Higher tau SUVR in cortical endpoints was associated with lower tractwise GFA (*p* <0.0001); and we observed higher GFA in tracts whose endpoints had greater SUVR differences (Figure 3).

**Conclusion:**

We replicated findings of lower WM connectivity in non‐amnestic than amnestic AD and showed WM integrity was associated with tau burden and atrophy in tract endpoints. Results were consistent across syndromes with differing cognitive symptoms and disease distributions.